# Comparative responses to demethylating therapy in animal models of osteosarcoma

**DOI:** 10.21203/rs.3.rs-4451060/v1

**Published:** 2024-06-11

**Authors:** Shan Huang, Ling Ren, Jessica A. Beck, Sushant Patkar, Maria Angeles Lillo Osuna, Aswini Cherukuri, Christina Mazcko, Susan A. Krum, Amy K. LeBlanc

**Affiliations:** National Cancer Institute, NIH; National Cancer Institute, NIH; National Cancer Institute, NIH; National Cancer Institute, NIH; University of Tennessee Health Science Center; National Cancer Institute, NIH; National Cancer Institute, NIH; University of Tennessee Health Science Center; National Cancer Institute, NIH

**Keywords:** Osteosarcoma, decitabine, estrogen receptor alpha, canine

## Abstract

**Background:**

The demethylating agent decitabine (DAC) effectively inhibits tumor growth and metastasis by targeting ESR1 methylation to restore estrogen receptor alpha (ERα) signaling and promoting cellular differentiation in models of human osteosarcoma (OSA). Whether this pathway can be targeted in canine OSA patients is unknown.

**Methods:**

Canine OSA tumor samples were tested for ERα expression and ESR1 promoter methylation. Human (MG63.3) and canine (MC-KOS) OSA cell lines and murine xenografts were treated with DAC *in vitro* and *in vivo*, respectively. Samples were assessed using mRNA sequencing and tissue immunohistochemistry.

**Results:**

ESR1 is methylated in a subset of canine OSA patient samples and the MC-KOS cell line. DAC treatment led to enhanced differentiation as demonstrated by increased ALPL expression, and suppressed tumor growth *in vitro* and *in vivo*. Metastatic progression was inhibited, particularly in the MG63.3 model, which expresses higher levels of DNA methyltransferases DNMT1 and 3B. DAC treatment induced significant alterations in immune response and cell cycle pathways.

**Conclusion:**

DAC treatment activates ERα signaling, promotes bone differentiation, and inhibits tumor growth and metastasis in human and canine OSA. Additional DAC-altered pathways and species- or individual-specific differences in DNMT expression may also play a role in DAC treatment of OSA.

## Background

Osteosarcoma (OSA) is a rare and aggressive pediatric/adolescent/young adult primary bone malignancy, but a more common cancer in large breed dogs. Clinically, the disease exhibits a high metastatic rate regardless of treatment type. Currently, the 5-year survival rate is less than 70% for humans[1], while the three-year survival rate for dogs is less than 10%[2]. OSA is characterized by a chaotic genome, with a high incidence of structural variations rather than druggable driving mutations in specific genes[3–5]. Both species have experienced little improvement in clinical outcomes over the past over 30 years[6, 7], underscoring the need for novel approaches to OSA therapy. Canine OSA shares many similarities with human disease, including gene expression, tumor biology, clinical features[8–10], and provides unique opportunities to test promising therapies for humans in comparative oncology clinical trials[10–13].

Estrogens contribute to skeleton growth and life-long bone homeostasis by acting on multiple cell types through a complicated signaling network[14]. They exert physiological functions in almost every tissue in the body in both males and females[15] by interacting with either estrogen receptors alpha (ERα) or beta (ERβ)[14, 16]. Both receptors are well expressed in bone marrow stromal cells[17, 18] and during osteoblast differentiation[19], but ERα mRNA was not detectable in human OSA cell lines HOS-TE85, SaOS2 and MG63[20]. Another study of 28 human OSA patient samples also showed no ERα expression by IHC[21]. A recent publication demonstrated that ERα was epigenetically silenced in several human OSA cell lines through promoter hypermethylation. DNA methylation changes are also widely seen in cancer cells[22, 23]. Multiple genes relevant to OSA progression[24] have been shown to exhibit inactivation due to aberrant DNA methylation in OSA human patient samples and cell lines, such as SPRY2[25], CXCL12[26], and NNAT[27]. Decitabine (DAC; 5-aza-2’-deoxycytidine) is a cytidine analog. It can incorporate into DNA strands during replication and irreversibly binds to DNA methyltransferase (DNMT) to reduce the levels of DNA methylation in a division-dependent manner thus restoring gene expression[28]. Critically, by utilizing the non-selective DNMT inhibitor DAC to restore ERα expression, OSA growth and metastasis have been significantly suppressed in a human xenograft model[29]. These findings formed the basis for the work shown herein, which leverages a comparative approach through the examination of canine OSA as a naturally occurring model of human OSA to support studies of biology and drug development.

## Methods

### Cell culture studies

Human MG63.3[30] and canine MC-KOS[31] OSA cells were grown at 37°C in 5% CO_2_ in DMEM supplemented with 10% fetal bovine serum, 2 mM glutamine, and Pen/Strep. Both cell lines were transfected with lentiviral green fluorescent protein (GFP) constructs (pSICO-eGFP or p960-X1–685-eGFP). Decitabine (Dacogen) was purchased from Otsuka (NDC 59148–046-70).

### Cell proliferation assays

Cell proliferation assays were performed using the IncuCyte ZOOM system (Essen BioScience Inc). Serial phase-contrast images were gathered and processed as the percentage of confluency to measure cell proliferation. Each data point represents the mean reading from sextuplicate analyses. All assays were conducted in duplicate. For drug treatment, the drug concentrations are indicated within the [1]s and text. Dimethyl sulfoxide (DMSO) was used as the control for group comparisons. EC_50_ was calculated using IncuCyte software using the data collected at 7 days post treatment.

#### In vivo orthotopic xenograft tumor growth and treatment

All animal work was conducted with the approval of the Animal Care and Use Committee of the National Cancer Institute under Animal Study Protocol PB-038. Primary tumor growth was evaluated by orthotopic injection of 10^6^ MG63.3 or MC-KOS cells/ 0.1 ml of Hank’s balanced salt solution (HBSS) into a parosseous site deep in the left caudal gastrocnemius of 6-week-old female SCID-Beige mice (Fox Chase CB17.B6-*Prkdc*^*scid*^*Lyst*^*bg*^/Crl) as described previously[32]. Treatment was initiated 10 days after tumor cell injection when primary tumors became palpable. For spontaneous metastasis model experiments, tumor bearing limbs were surgically amputated on Day 21, with treatment initiation 3 days after amputation. Experimental metastasis experiments were used for the MC-KOS cell line due to the protracted period of time needed for development of spontaneous metastases. 10^6^ MC-KOS cells/ 0.1 ml of HBSS was injected through tail vein, and the treatment was started the day after cell injection. Mice received intraperitoneal injection (ip) of sterile saline (Control) and DAC (1mg/kg) on Monday-Wednesday-Friday with 2-week on 1-week off schedule. After 25 days, five mice from each group were euthanized to assess lung metastasis burden. The remaining mice were used for survival analysis. The volume of orthotopic tumor growth was measured once a week with digital calipers to obtain two diameters of the tumor sphere determined using the equation (*D × d*^*2*^)/6 × 3.12 (where *D* = the maximum diameter and d = the minimum diameter).

### Pulmonary metastasis assessment

Early micro-metastases of lungs were examined and imaged with fluorescence inverted microscope (Leica DM IRB) with an attached CCD camera. Whole lung images were taken using a Leica MZ FLIII fluorescence stereomicroscope. The areas of fluorescent lung metastases were quantified with ImageJ software. Lung metastases were also examined using H&E-stained paraffin-embedded sections.

### Canine tissue samples

Treatment-naïve canine OSA tumor and normal bone samples were collected at the time of therapeutic surgical limb amputation from patients enrolled in the NCI Comparative Oncology Trials Consortium (COTC) 021/022 trials, as described previously[12].

### Methylation specific PCR and bisulfite sequencing

Total cellular DNA was extracted from cells by using DNeasy Blood & Tissue Kit (Qiagen) following the manufacturer’s recommendations. Each DNA sample was collected in triplicate. EpiMark Bisulfite Conversion Kit (New England Biolabs, Inc.) was used following the manufacturer’s recommendations. For PCR amplification of the methylated and unmethylated ESR1 promoter, PCR was performed on the bisulfite-converted DNA with the following primers: Methylated ESR1 F GAGTAGTTATAGTTACGGGGTCGTC, R AATTTTCTTCCTACTACCAAACGAA; Unmethylated ESR1 F AGTAGTTATAGTTATGGGGTTGTTG and R AATTTTCTTCCTACTACCAAACAA

The PCR products were then analyzed by agarose gel electrophoresis. For bisulfite sequencing the PCR products were cloned into pCRII with the TOPO-TA Cloning Kit (Life Technologies). Individual clones were sequenced by Sanger sequencing with the T7 promoter primer. The data were analyzed with BISMA[33]. Quantitative PCR Total cellular RNA was extracted from cells with TRIzol Reagent (Invitrogen). Each RNA sample was collected in biological triplicates and each qPCR reaction was amplified in triplicate. Total RNA was converted to cDNA with Maxima First Strand cDNA Synthesis Kit (ThermoFisher Scientific) according to the manufacturer’s instructions. cDNA was subjected to quantitative PCR using the Maxima SYBR Green qPCR Master Mix with ROX (ThermoFisher Scientific). Gene expression levels were compared after normalization to endogenous β-actin (Actb). Primers were selected using Primer-BLAST and the sequences are: Actb: TGTGTTATGTGGCCCTGGAC and TTCCATGCCCAGGAAGGAAG, and ESR1: GCATCCAGGGAAGCTCTTCTT and TCTCTTCCAGAGACTTCAGGGT, and ALPL: CCAAGGACGCTGGGAAATCT and ACGTTGTGCATGAGCTGGTA.

### Immunohistochemistry (IHC)

Formalin-fixed, paraffin-embedded murine tissues were immunolabeled for estrogen receptor α (ERα, abcam ab259427, 1:400), alkaline phosphatase (ALP, abcam ab65834, 1:250), osteomodulin (OMD, abcam ab154249, 1:400), Sp7/Osterix (abcam ab209484, 1:400), and SOX2 (abcam ab97959, 1:800) by VitroVivo Biotech. The H-score was quantified in annotated lung metastases using HALO’s cytonuclear algorithm, which captures both the intensity and the proportion of the biomarker of interest from the IHC image.

### Alkaline phosphatase staining

MC-KOS cells were treated with 20 μM DAC for 72 hours and then fixed with 3.7% formaldehyde and stained for alkaline phosphatase using SIGMAFAST BCIP/NBT (Sigma-Aldrich).

### Nucleic acid isolation and mRNA sequencing

MG63.3 and MC-KOS cells treated with DAC and DMSO *in vitro* for 24 hours were collected, with 3 replicates for each group. mRNA was isolated from cell line pellets using Qiagen Allprep DNA/RNA Mini Kit (Cat#80204). The total mRNA quality and quantity were assessed using Nanodrop 8000 (ThermoFisher) and Agilent 4200 Tapestation with RNA Screen Tape (Cat# 5067–5576) and RNA Screen Tape sample Buffer (Cat#5067–5577). All samples forwarded for mRNA sequencing had a RIN > 8 and a total RNA quantity > 100 ng.

Canine MC-KOS mRNA-Seq samples were pooled and sequenced on NovaSeq 6000 S2 using Illumina^®^ Stranded mRNA Prep and paired-end sequencing. The samples have 136 to 185 million pass filter reads with more than 90% of bases above the quality score of Q30. Reads of the samples were trimmed for adapters and low-quality bases using Cutadapt before alignment with the reference genome (GSD_1.0, also referred to as canfam4)[34] and the annotated transcripts using STAR. The average mapping rate of all samples was 95%, with unique alignment above 75%. There were 3.35 to 10.99% unmapped reads. The mapping statistics were calculated using Picard software. The samples have 3.14% ribosomal bases. Percent coding bases are between 49–60%. Percent UTR bases are 24–28%, and mRNA bases are between 76–87% for all the samples. Library complexity was measured in terms of unique fragments in the mapped reads using Picard’s MarkDuplicate utility. The samples had 68–76% non-duplicate reads.

Human MG63.3 mRNA-Seq samples were pooled and sequenced on NovaSeq 6000 S2 using Illumina^®^ Stranded mRNA Prep and paired-end sequencing. The samples had 105 to 159 million pass filter reads with more than 91% of bases above the quality score of Q30. Reads of the samples were trimmed for adapters and low-quality bases using Cutadapt before alignment with the reference genome (hg38) and the annotated transcripts using STAR. The average mapping rate of all samples is 93%, with unique alignment above 85%. There were 5.25 to 8.72% unmapped reads. The mapping statistics were calculated using Picard software. The samples have 0.75% ribosomal bases. Percent coding bases are between 57–63%. Percent UTR bases are 31–37%, and mRNA bases are between 94–95% for all the samples. Library complexity was measured in terms of unique fragments in the mapped reads using Picard’s MarkDuplicate utility. The samples had 59–72% non-duplicate reads.

### Differential expression and gene set enrichment analysis

Raw read count data from untreated and DAC-treated osteosarcoma cell lines are provided as input to edgeR[35] (v3.40.2) using default parameter settings for differential expression analysis. This analysis was done independently for both canine and human osteosarcoma cell lines. Following differential expression analysis, all genes were ranked by their log fold change in expression estimated by edgeR. Genes ranked at the top of the list with high positive log fold change correspond to genes that had relatively higher expression in DAC-treated compared to untreated cell lines, whereas genes with high negative log fold change in expression had relatively lower expression in DAC treated compared to untreated cell lines. The ranked list of all genes based on the log-fold change was then provided as input to the standard Gene Set Enrichment Analysis (GSEA) pipeline implemented in clusterProfiler[36] (v4.6.2) with the following parameters specified: (nPermSimple: 100000, minGSSize = 10, maxGSSize = 500) to estimate the relative enrichment of cancer hallmark pathways in DAC treated and untreated cell lines.

### Visualization of gene expression data in heatmaps

Normalized read count data (TPM) for MC-KOS and MG63.3 osteosarcoma cell lines were log2 transformed and scaled to obtain Z scores. The Z scores are then visualized in heatmaps as shown in Fig.5.

### Statistical analysis

Unpaired T-test was used to compare the difference between the groups using GraphPad 10.0.3. Data within the figures are presented as mean ± standard error of the mean. Pathway enrichment p-values were estimated using the hypergeometric test. P-values and all pathways with false discovery rates (FDR) < 0.05 were considered significant.

## Results

### DAC inhibits human OSA growth and metastasis

**Figure 1:**
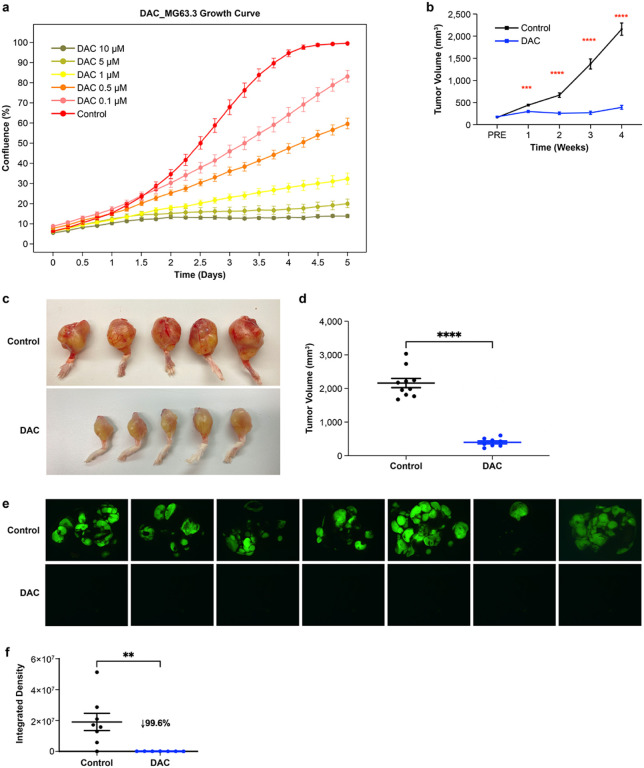
Decitabine (DAC) therapy inhibits human OSA growth and metastasis **a** *In vitro*growth inhibition of MG63.3 human OSA cells (initial plating of 500 cell/well) over a range of decitabine exposures, exhibiting a dose-response relationship. MG63.3 vehicle control is depicted by the red curve. **b** *In vivo*growth inhibition of MG63.3 xenografted primary tumors with decitabine therapy. **c** Depiction of MG63.3 gross primary tumor sizes at experimental endpoint, with and without DAC therapy. **d** Quantification of MG63.3 primary tumor size measurements, with and without DAC therapy. **e**Representative fluorescent stereomicroscopy images of MG63.3 lung metastasis, with and without DAC therapy. **f** Fluorescence quantification of lungs depicted in Fig. 1e. ** p<0.01; *** p<0.001; **** p<0.0001

DAC treatment was first tested on the highly metastatic human OSA cell line MG63.3. MG63.3 was sensitive to DAC (Fig.1a), with a half maximal effective concentration (EC_50_) = 0.66 μM at Day 7 (Fig.S1a). Primary tumor growth *in vivo* was significantly slowed after one week of DAC treatment (Fig.1b), with minimal increase over the four-week regimen (Fig.1c-d). Lungs were collected and observed under fluorescence microscopy to identify and quantify GFP-positive pulmonary metastases. Compared to the control group which developed multiple lung metastatic nodules, DAC-treated mice developed fewer pulmonary micro-metastases (Fig. S1b). To study DAC effect on early lung metastasis, another primary tumor model group was generated (Fig. S1c) which developed early lung metastasis by Day 19 (Fig. S1d) followed by surgical limb amputation (Day 21) and DAC treatment (Day 24). After 45 days, this early metastasis model also identified significantly fewer metastases in DAC-treated mice showed compared to control (Fig.1e-f) underscoring the effectiveness of DAC on metastatic progression.

### The ESR1 promoter is methylated in canine OSA samples

**Figure 2:**
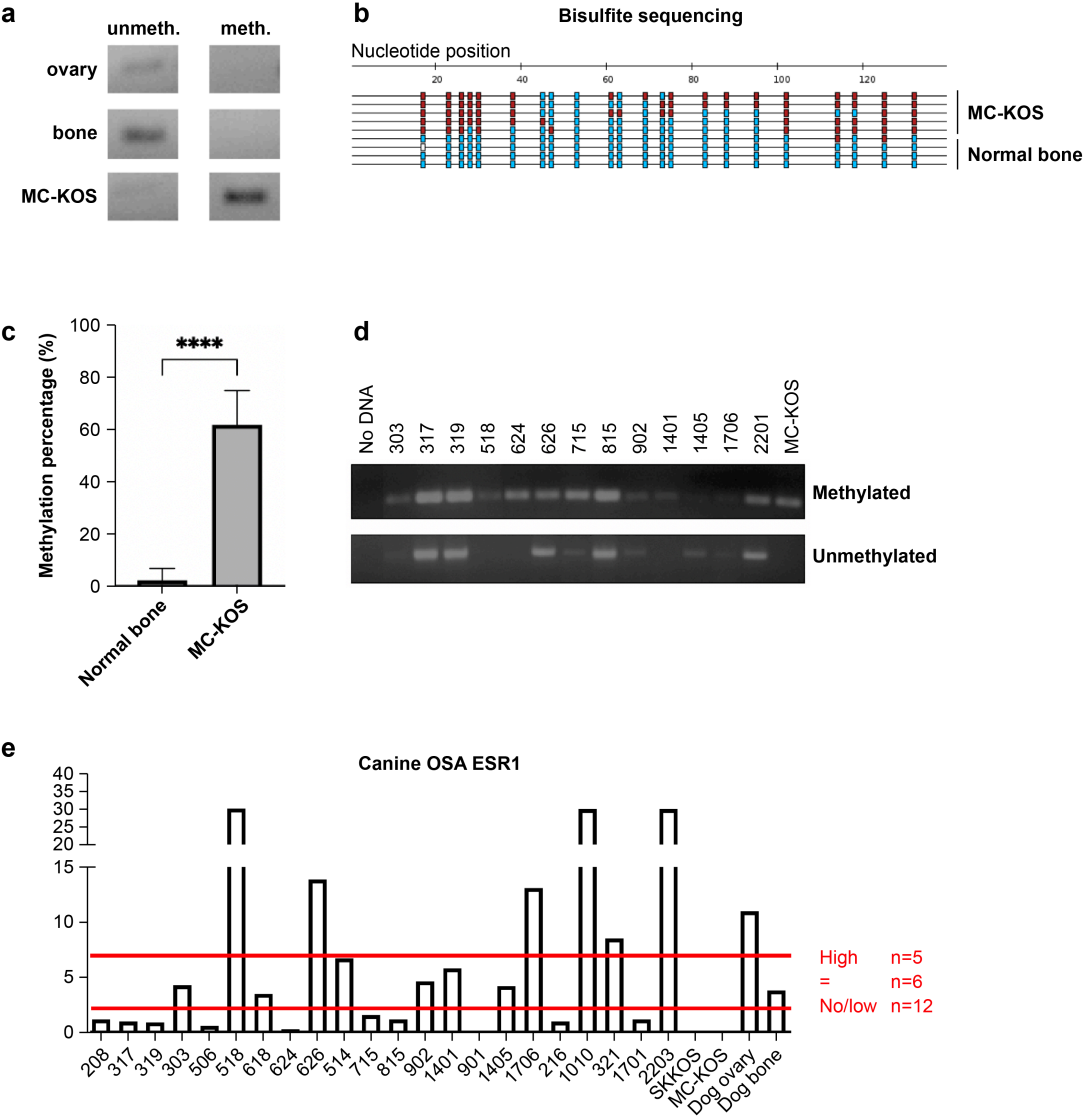
ESR1 is methylated in canine osteosarcoma **a** Comparison of ESR1 methylation status via methylation-specific PCR between normal canine bone, normal canine ovary, and the OSA cell line MC-KOS. **b** Comparative location mapping of methylation of the ESR1 promoter determined through bisulfite sequencing. **c** ESR1 methylation quantification of Fig. 2b. **d** ESR1 methylation status of canine OSA patients’ primary tumor samples and the MC-KOS cell line. **e** Relative quantification (RQ) of ESR1 expression by mRNA in canine patient osteosarcoma samples (represented by patient ID number on the x-axis). **** p<0.0001

The effects of DAC in human osteosarcoma have been in part attributed to its demethylating effects on the ESR1 promoter in human tumors[29]. Little is known about the role and status of ESR1 in canine osteosarcoma. Methylation-specific PCR was performed on normal canine bone and the MC-KOS canine OSA cell line to assess methylation of the ESR1 promoter region (Fig.2a). Normal canine ovarian tissue was also tested as a positive control for unmethylated DNA, as there is high expression of ESR1. The specific methylation locations at ESR1 promoter region were explored using bisulfite sequencing (2b). The methylation percentage of the canine OSA cell line (MC-KOS) was significantly higher than normal bone tissue at approximately 60% (2c). Finally, 15 canine OSA patients’ tumor samples were examined for ESR1 promoter methylation (2d). Five (33%) presented complete methylation; seven (47%) were partial; only one sample was totally unmethylated. ESR1 expression varied in canine osteosarcomas but was low to undetectable in over half of the samples (n = 12; Fig. 2e).

### DAC restores ERα and promotes cellular differentiation of canine OSA

**Figure 3:**
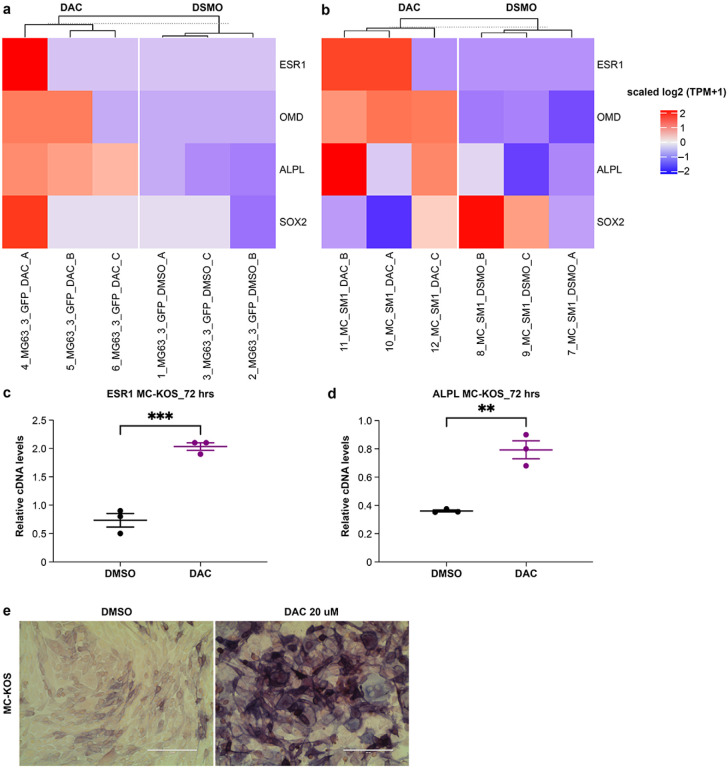
DAC restores ERα and promotes cellular differentiation of canine OSA **a** Gene expression heatmap demonstrating increased expression of ESR1 and bone mineralization markers in control and DAC treated MG63.3 and **(b)** MC-KOS cell lines. **c** Relative cDNA levels of ESR1 and **(d)** ALPL after 72 hrs DAC treatment in MC-KOS. **e** Alkaline phosphastase staining in control and DAC-exposed MC-KOS cells (72 hrs). ** p<0.01; *** p<0.001

Since a subset of canine patients demonstrate ESR1 hypermethylation (Fig. 2) similar to that described in human OSA patients, we next investigated DAC treatment in canine OSA cells to determine whether they respond similarly to human OSA cells[29] including restoration of ERα and enhanced cellular differentiation. Canine and human OSA cells were exposed to DAC *in vitro* and collected after 24 hours for mRNA sequencing. Markers of bone mineralization, alkaline phosphatase (ALPL) and osteomodulin (OMD), were increased in MG63.3 (logFC = 3.21, FDR < 0.0001) and MC-KOS (logFC = 1.51, FDR < 0.0001) respectively (Fig. 3a–b). Although not significantly different at this timepoint, ESR1 mRNA levels of both cell lines were slightly increased after 24-hour treatment with DAC, with logFC = 2.70 and 1.59 for MG63.3 and MC-KOS respectively. After 72 hours, canine OSA cells treated with DAC demonstrated increased ERα cDNA (Fig. 3c) which was associated with increased expression and activity of the bone differentiation marker ALPL *in vitro* (Fig. 3d–e).

### DAC inhibits canine OSA growth and metastasis

**Figure 4:**
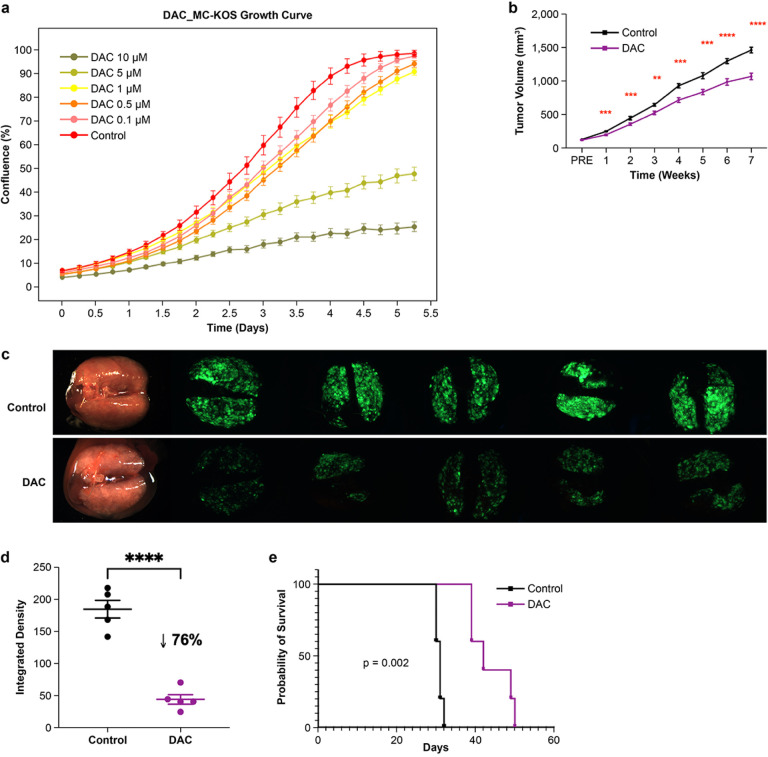
DAC inhibits canine OS primary tumor growth and metastasis **a** *In vitro* growth inhibition of MC-KOS canine OSA cells (initial plating of 500 cell/well) over a range of decitabine exposures, exhibiting a dose-response relationship. MC-KOS vehicle control is depicted by the red curve. **b** Modest *in vivo* growth inhibition of MC-KOS xenografted primary tumors with decitabine therapy. **c**Depiction of MC-KOS gross and fluorescent stereomicroscopy images of experimental lung metastases with and without DAC therapy, with quantification of fluorescent images given in panel **(d)**. **e** Kaplan-Meier survival curve of MC-KOS experiment metastasis mice treated with DAC. ** p<0.01; *** p<0.001; **** p<0.0001

We next aimed to investigate the effects of DAC treatment on canine OSA progression. *In vitro* cell proliferation testing showed DAC suppressed MC-KOS growth (4a) with an EC50 = 4.69 μM at Day 7 (Fig. S2a). *In vivo*, primary tumor growth inhibition was observed as early as one week after treatment, but in contrast to MG63.3(Fig. 4b, Fig. S2b), the effect did not amplify much over time for MC-KOS. We next aimed to investigate the effects of DAC on metastasis, the primary determinant of prognosis in canine and human OSA patients[1, 7]. In an experimental metastasis model, DAC-treated mice developed significantly fewer metastases than the control group (Fig. 4c–d, p < 0.0001). The median survival for control and DAC-treated groups were 31 and 42 days respectively (p = 0.002, Fig.4e). Although the differences were minimal, increased immunolabeling of ERα, ALP, OMD, Sp7, SOX2 was observed in DAC-treated lung metastases *in vivo* (Fig. S2c); no differences in immunoexpression were identified between DAC-exposed and control primary tumors (PT).

#### DAC exposure in human and canine OSA leads to significant alterations in pathways including immune function and cell cycle progression

**Figure 5:**
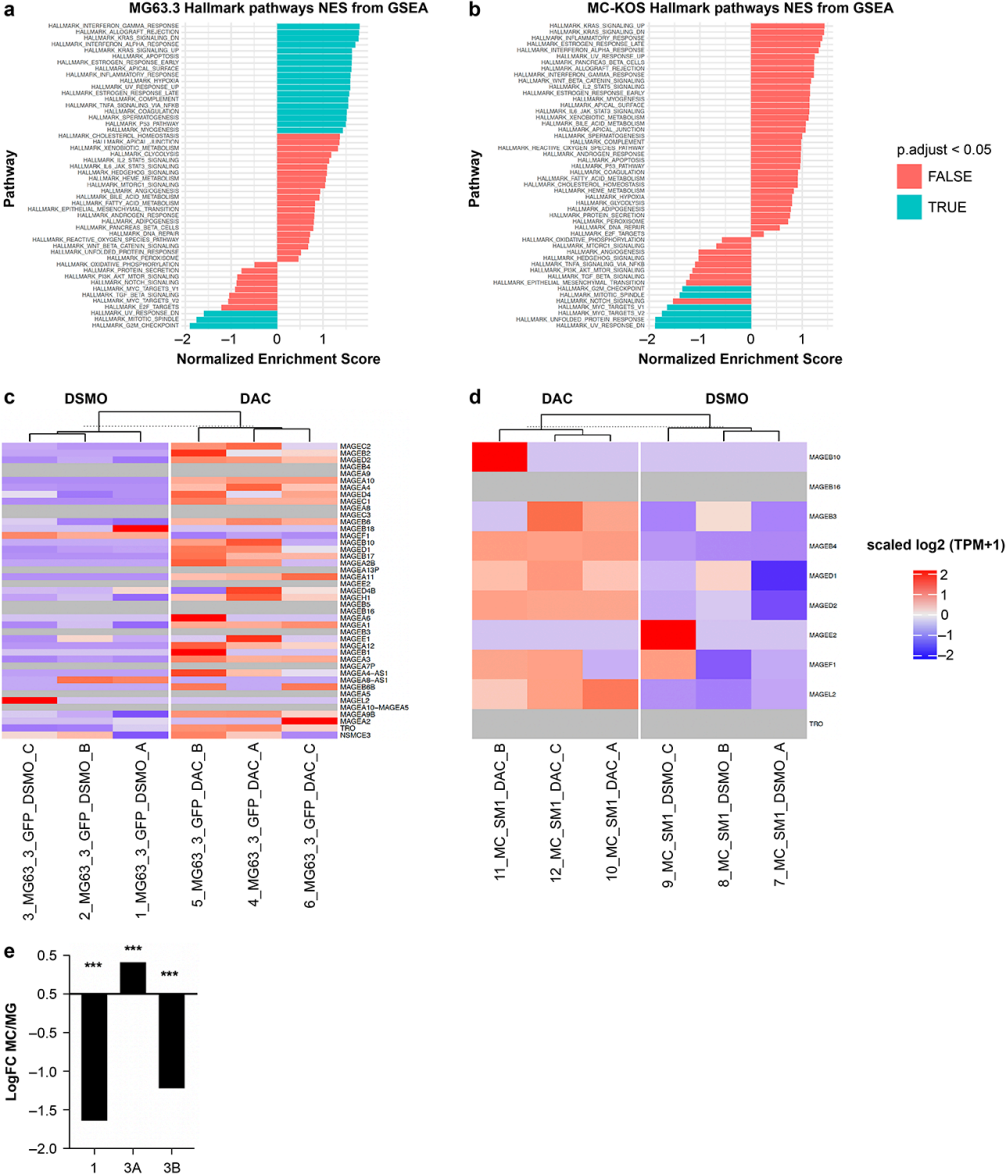
DAC exposure in human and canine OSA leads to significant alterations in pathways including immune function and cell cycle progression Gene set enrichment analysis (GSEA) of **(a)** MG63.3 and **(b)** MC-KOS cells after DAC treatment. Gene expression changes of MAGE family in **(c)**MG63.3 and **(d)** MC-KOS. **e** DNMTs expression level given as log Fold Change (FC) of MC-KOS / MG63.3. MC = MC-KOS, MG = MG63.3. *** p<0.001

DAC exposure restores ESR1 in canine OSA cells (Fig. 3c); however, DAC is a non-specific demethylating agent which may have additional anti-tumor activities. Gene set enrichment analysis (GSEA) showed more significantly altered pathways following DAC exposure in MG63.3 cells (Fig. 5a) compared to MC-KOS (5b). These early alterations included activation of immune/inflammation-related pathways, and activation or suppression of cell cycle relevant mechanisms. Because we identified immune-related pathways, we next aimed to interrogate the Melanoma Antigen Gene (MAGE) family which is composed of cancer biomarkers and targets of immunotherapies[37, 38]. Multiple genes in this family showed greatly increased mRNA expression after DAC treatment in MG63.3 and MC-KOS (Fig. 5c–d). Alterations in other genes with known association with OSA such as NNAT and CXCL12 demonstrated favorable changes under DAC treatment (Table S1). Finally, although we were able to demonstrate that DAC was effective in both canine and human OSA models, we identified increased effectiveness of DAC in MG63.3 *in vitro* (Fig. S1a, S2a, S2b) and in metastatic progression *in vivo* compared to MC-KOS (Fig. 1f, Fig. 4d). Based on these data, we investigated the expression of DAC’s target, DNMT, in the MG63.3 and MC-KOS cell lines. Compared to MG63.3, MC-KOS had significantly lower expression of DNMT1 and DNMT3B, but a higher level of DNMT3A (Fig. 5e).

## Discussion

ESR1 has been shown to be hypermethylated in human OSA. Use of the DNMT inhibitor DAC restores ERα expression with subsequent inhibition of OSA growth and metastasis in three human OSA cell lines[29]. These findings provided rationale for our experiments in a fourth human cell line (MG63.3). DAC-treated MG63.3 demonstrated reduced primary tumor growth and significantly fewer pulmonary metastases. After confirming DAC’s activity in human OSA, our study focused on the comparative value of DAC in canine OSA. Canine OSA presents a favorable patient model for drug testing due to its clinical, pathological, and molecular similarities to human OSA[8–10]. Bolstered by the higher annual incidence of OSA in dogs, the canine model is also a rational bridge to future comparative oncology trials conducted in pet dogs with naturally occurring OSA[10–13].

Using the canine patient-derived MC-KOS cell line[31], DAC was demonstrated to inhibit tumor growth and metastasis in canine OSA. When compared, DAC decreased MG63.3 spontaneous metastasis more effectively (99.6%) compared to MC-KOS experimental metastasis (reduced by 76%). This difference may suggest that DAC is more effective in the spontaneous metastasis setting. Alternatively, differences in the DAC-targeted DNMTs were also observed. Namely, MG63.3 had higher expression of DNMT1 and 3B compared to MC-KOS. Future studies aimed at further investigating the roles of DNMTs in DAC treatment of canine OSA are warranted and should consider species and individual tumor differences in DNMTs expression.

There is a paucity of data on estrogen signaling in canine OSA. Using patient tumor samples derived from an NCI-sponsored canine clinical trial[12], ESR1 was examined. Hypermethylation of ESR1 was observed in a subset of canine OSA patients. DAC treatment of MC-KOS was associated with increased ESR1 and enhanced cellular differentiation. This is consistent with previous studies in human OSA[29] and underscores a shared pathway that could be targeted therapeutically. Interestingly, the finding of downregulated SOX2 after 24 hours of exposure to DAC *in vitro* was also consistent with the previous study[29]. But the importance of elevated SOX2 immunoexpression in metastases is unclear. This may be due to non-specific demethylation by DAC. Additional experimental work to better characterize cells that form early-stage micrometastases in DAC-treated mice is warranted.

Prior research has provided strong evidence that the restoration of ESR1 in human OSA cell lines significantly inhibits tumor progression[29]. But since DAC is a non-selective DNMT inhibitor, affecting broadly on numerous genes, it remains possible that other pathways/genes are impacted as well. By collecting cell samples 24h after DAC treatment, we were able to observe a window of time before ERα expression is fully restored. Several pathways and bone mineralization markers were already significantly altered at this timepoint. In addition, GSEA revealed enrichment in the MG63.3 treated group of pathways involved in innate immunity and inflammation, such as interferon response and complement system, suggesting the importance of immune processes in disease progression. These immune based gene signatures have also been reported in other human and canine OSA studies with enrichment associated with better disease outcomes[13, 39]. The MAGE family Cancer-testis antigens (CTAs) are primarily expressed in testicular germ cells and placental tissue, but aberrantly present on a wide variety of solid tumors due to CpG islands demethylation[38]. These proteins are immunogenic, especially for A10[40] and A3[41, 42], and are potential targets for cancer immunotherapy. Our data indicates that DAC effectively boost MAGE expression, which is consistent with findings described elsewhere[43–45]. It is possible that clinical use of DAC in conjunction with other immune therapies could result in improved outcomes and are rational candidate strategies for future studies[43–45].

## Conclusions

DAC treatment reduces primary tumor growth in canine and human OSA models. The development of pulmonary metastases was dramatically reduced in both models suggesting that metastatic lesions may be more vulnerable to epigenetic treatment. Although DAC significantly reduced canine OSA growth, the effects were more significant in the MG63.3 human OSA model which has higher expression of DAC-targeted DNMTs compared to MC-KOS. Finally, in addition to its effects on ER signaling, multiple pathways including immune activation are altered following DAC treatment. Future studies investigating the impact of DAC on DNMTs and immune pathways in canine OSA models are warranted.

## Data Availability

Data is provided within the manuscript or supplementary information files. mRNA sequence data generated in this study will be deposited into the Gene Expression Omnibus (GEO) prior to manuscript publication.
